# Acknowledgement to Reviewers of *Biology* in 2017

**DOI:** 10.3390/biology7010011

**Published:** 2018-01-11

**Authors:** Biology Editorial Office

**Affiliations:** MDPI AG, St. Alban-Anlage 66, 4052 Basel, Switzerland

Peer review is an essential part in the publication process, ensuring that *Biology* maintains high quality standards for its published papers. In 2017, a total of 50 papers were published in the journal. Thanks to the cooperation of our reviewers, the median time to first decision was 24 days and the median time to publication was 64 days. The editors would like to express their sincere gratitude to the following reviewers for their time and dedication in 2017:
Adam, PaulLeoni, BarbaraAmbrose, R.P. KingslyLevin, MichaelAnifandis, GeorgeLewis, Jesse S.Archer, SimonLi, CongjunArredondo, Luis José DelayeLi, ZhihengBalestrieri, RosarioLigon, Day B.Becker, Donald F.Longo, GiuseppeBilbao, Jose RamonLopez, Dolores E.Bjork, BryanLuo, ShuaiBlackstone, NeilLutz, StefanieBoczek, NicoleMaiato, HelderBohutskyi, PavloMallo, MoisesBorger, PieterMartin, BradleyBozzaro, SalvatoreMazzulli, JoeBrand-Saberi, BeateMc Auley, Mark T.Braun, Edward L.McGuinness, DagmaraBricknell, IanMcKim, Kim S.Brown, Robert J.McLoughlin, GráinneBrownstein, Zippora (Zippi)Mehrshahi, PayamBrunetti-Pierri, NicolaMenger, FredricCampos, FranciscoMiller, Robert H.Catacuzzeno, LuigiMinenkova, OlgaChain, Patrick S.G.Møller, AndersChandaka, Giri KumarMulero, VictorianoChen, Bor-SenMüller, PeterChen, JinfengMuller, UlrichCheon, Yong-PilNavarro, PilarChiao, Ying AnnNawrot, RobertChristina, SpryNemec, Antonia A.Cimini, DanielaNeumann Andersen, MathiasColley, Karen J.Nishimura, Shin-ichiroConstantinescu, StefanNoble, DenisCreixell, Teresa AdellPaldi, AndrasCurtale, GraziellaPalles, ClaireDe Martino, AndreaPandhal, JagroopDen Hertog, JeroenPanico, AntonioDimitrakopoulos, Georgios N.Peracchi, AlessioDu, MinPerego, CarlaDurston, AntonyPesole, GrazianoEllis, Ronald E.Petro, Thomas M.Fabre, PierrePitto, LetiziaFainsod, AbrahamPonnambalam, SreenivasanFalconer, RobertPritchard, Hugh W.Fernández-Méndez, MarRamos-Suárez, Juan LuisFigueras Huerta, AntonioRe, LambertoFracasso, AlessandraReschen, Michael EdwardFuentes, AugustínRijkers, GerFunk, RichardRisk, Michael J.Gales, AmandineRizos, DimitriosGannon, Victor P.J.Roach, DwayneGazouli, MariaRoig Molina, Francisco JoseGebhardt, ChristofRoli, AndreaGlazier, Douglas S.Roos, Wynand P.Gong, ShanzhongRutishauser, Urs StephenGreen, Sara Marie EhrenreichSalinas, IreneGualtieri, PaoloSalmon, Edward D.Hantusch, BrigitteSauerwein, HelgaHarduin-Lepers, AnneSchibler, UeliHejnol, AndreasSchmidt-Ott, UrsHiroi, NoboruSeligmann, HervéHo, SamuelShumskaya, MariaHuang, Yen-LinSigurdsen, TrondIgamberdiev, AbirSitja-Bobadilla, AriadnaIshikawa, TakahiroSmith, RobertIto, YujiSpanò, StefaniaJain, ShaluStacewicz-Sapuntzakis, MariaJay Ko, ChemYongStaehle, MaryJin, FanStockan, JenniJühling, FrankStone, Trevor WilliamKaneko, GenSu, TaoKanno, YuichiroSuzuki, Yuichiro J.Kaski, Juan PabloSyrén, Per-OlofKavaliers, MartinSzekely, GyorgyKawakami, JunjiTaylor, AndrewKawashima, HirotoTorinsson Naluai, ÅsaKhisamutdinov, EmilTosic, PredragKieber-Emmons, ThomasTraer, ElieKiljunen, SaijaTsounis, GeorgiosKim, JongpilVarma, DileepKondo, JiroVisintainer, RobertoKorstad, JohnWang, KaiKoslicki, DavidWebb, GrahameKoslicki, DavidWenger, AmeliaKrämer, Oliver H.Wichterle, HynekKunkle, BrianWinuthayanon, WipaweeLackie, Peter M.Witte, WolfgangLafont, FrankYUEN, Karen Wing YeeLange, MilesZammit, CarlaLei, LeiZiermann, Janine

